# Tailoring grain boundary resistance in Li-ion conducting polymer–ceramic hybrid electrolytes based on polyether and Li_1.5_Al_0.5_Ge_1.5_(PO_4_)_3_

**DOI:** 10.1039/d5ra07453c

**Published:** 2025-10-29

**Authors:** Naamo Suzuki, Koji Hiraoka, Koji Ohara, Kenta Fujii, Shiro Seki

**Affiliations:** a Graduate School of Applied Chemistry and Chemical Engineering, Kogakuin University 2665-1 Nakano-machi Hachioji Tokyo 192-0015 Japan shiro-seki@cc.kogakuin.ac.jp; b Japan Synchrotron Radiation Research Institute/SPring-8 Sayo-cho Sayo-gun Hyogo 679-5198 Japan; c Faculty of Materials for Energy, Shimane University Matsue Shimane 690-8504 Japan; d Department of Applied Chemistry, Yamaguchi University Ube 755-8611 Japan

## Abstract

Composite solid electrolytes comprising ceramic and polymer components have garnered significant attention as promising materials for next-generation all-solid-state lithium batteries owing to the combination of high ionic conductivity and enhanced interfacial stability. In this study, we systematically investigate the effects of incorporating either crystalline or amorphous Li_1+*x*_Al_*x*_Ge_2−*x*_(PO_4_)_3_ (LAGP) into a polyether-based polymer matrix. Differential scanning calorimetry reveals that the addition of LAGP does not markedly influence the thermal transitions of the host polymer, suggesting minimal disruption of polymer chain dynamics. Ionic conductivity measurements indicate that crystalline LAGP slightly reduces overall conductivity, whereas amorphous LAGP effectively mitigates the conductivity drop at lower temperatures, potentially providing alternative Li^+^ conduction pathways through the amorphous phase. Impedance spectroscopy shows significant grain boundary resistance in composites with crystalline LAGP, whereas those with amorphous LAGP exhibit improved interfacial ion transport, particularly under non-blocking electrode conditions. High-energy X-ray diffraction using synchrotron radiation and pair distribution function analysis further confirms homogeneous structural integration between the polymer and amorphous LAGP. These findings demonstrate that the microstructure of ceramic fillers, particularly their amorphous nature, plays a pivotal role in dictating ion transport behavior, providing valuable insights for the design of high-performance composite electrolytes.

## Abstract 2

Polyether/Li_1+*x*_Al_*x*_Ge_2−*x*_(PO_4_)_3_ hybrid electrolytes were investigated to assess the effect of inorganic crystallinity, and exhibited low-temperature ionic conductivity and reduced grain boundary resistance.

## Graphical abstract

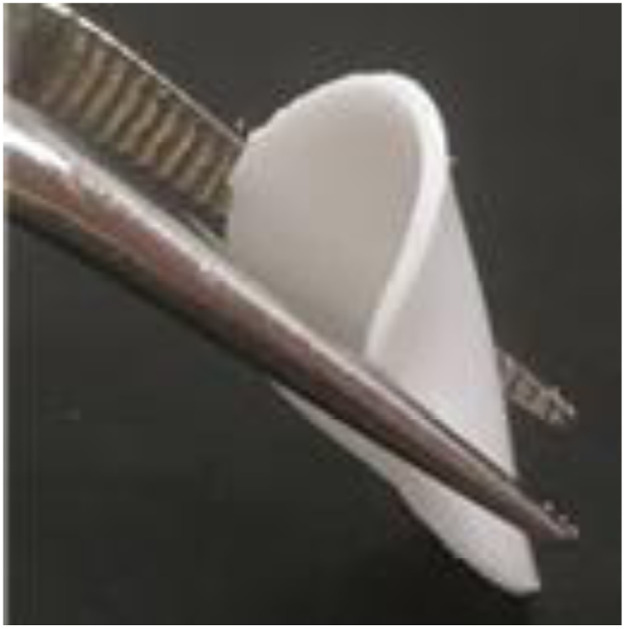

## Introduction

The transition toward a low-carbon society and the escalating global demand for energy underscore the urgent need to minimize environmental impacts through more efficient energy utilization.^1^ In turn, the widespread integration of renewable energy sources requires advanced energy storage technologies capable of stabilizing power supply fluctuations.^2^ Among these technologies, lithium-ion batteries have garnered significant attention because of their high energy density and efficiency; consequently, even though they have already been widely implemented in mobile devices, further performance improvements are anticipated.^3–5^ For large-scale energy storage applications, material safety is a critical factor, and in this regard,^6^ all-solid-state batteries (ASSBs) employing solid electrolytes have emerged as promising candidates.^7^ ASSBs offer several advantages over conventional liquid-electrolyte-based batteries, including enhanced safety, owing to the elimination of flammable organic solvents and corrosive electrolytes, and the potential for simplified cell architectures. These features facilitate higher energy density through the removal of separators and the possibility of multilayer stacking within a shared enclosure.^8^ Solid electrolytes used in ASSBs are broadly classified into two categories: inorganic and polymer-based materials.^9^ Inorganic solid electrolytes include ceramics and glasses, with ceramics exhibiting a particularly distinctive property, namely ion-specific conduction. For instance, Li_0.34_La_0.51_TiO_2.94_ selectively conducts lithium ions,^10^ whereas Na_3_Zr_2_Si_2_PO_12_ (NZSP) selectively conducts sodium ions.^11^ This selectivity results in a transference number of unity, contributing to higher ionic conductivity. Moreover, the absence of solvent molecules suppresses side reactions at the electrode–electrolyte interface.^12^ Although some recent ceramic materials, such as Li_10_GeP_2_S_12_, have demonstrated high ionic conductivity (2.2 × 10^−3^ S cm^−1^ at 298 K),^13^ the overall conductivity of inorganic solid electrolytes remains lower than that of liquid electrolytes. Thin-film fabrication can reduce resistance, but poor mechanical strength, interfacial instability during cycling, and significant grain boundary resistance pose major challenges, especially in polycrystalline and sintered materials. The grain boundary resistance in such materials can be over an order of magnitude higher than the bulk resistance, as observed in Li_1.3_Al_0.3_Ti_1.7_(PO_4_)_3_ (LATP)-based systems.^14^

In contrast, polymer-based solid electrolytes achieve ionic conductivity through the segmental motion of polymer chains that host dissolved electrolyte salts. Despite their relatively low ionic conductivity (∼10^−6^ S cm^−1^) and limited lithium-ion transference numbers,^15^ these materials exhibit high mechanical flexibility, self-supporting film-formability, and interfacial compatibility. Among them, polyethylene oxide (PEO), which contains ether groups, is the most extensively studied. However, its tendency to crystallize hinders ion mobility. This limitation can be mitigated by copolymerization or blending with polymers such as polypropylene oxide.^16^ To leverage the advantages of both inorganic and polymer systems, polymer/inorganic composite solid electrolytes have gained increasing attention.^17,18^ These materials are expected to offer improved ionic conductivity, interfacial stability, and mechanical compliance. Our group has previously demonstrated the feasibility of such composite electrolytes in sodium-based [NaCoO_2_/Na] and [SPAN/Na] cells.^19^ However, in composite systems with high inorganic contents, the formation of continuous inorganic particle networks often introduces grain boundary resistance, resulting in decreased ionic conductivity.^20^ To address this issue, the present study explores the use of amorphous inorganic materials as a strategy to suppress grain boundary resistance in composite electrolytes. Considering that grain boundaries are intrinsic to crystalline structures, incorporating amorphous inorganic phases is expected to mitigate such interfacial resistance when hybridized with polymers. As a model material, we selected Li_1.5_Al_0.5_Ge_1.5_(PO_4_)_3_ (LAGP),^21^ which can be obtained in both amorphous and rhombohedral crystalline forms, thereby enabling direct comparison of the ion transport behaviour with and without grain boundaries. LAGP is derived from the NASICON-type structure,^22^ originally based on LiTi_2_(PO_4_)_3_ (LTP), and is known for its air stability.^23^ Compared with LATP, where Ti reacts unfavourably with lithium metal, leading to electron conduction *via* Ti^4+^/Ti^3+^ redox activity,^24^ LAGP^21,25^ offers improved interfacial stability with lithium anodes, by substituting Ti with Ge. Nevertheless, there may be some reactivity between Ge and lithium, which remains an open question.^26^ Notably, Y.-C. Jung *et al.* demonstrated that PEO/LAGP composite electrolytes can achieve ionic conductivities on the order of 1.0 × 10^−5^ S cm^−1^.^22^ Moreover, the incorporation of glass-ceramic LAGP particles has been shown to suppress PEO crystallization, thereby enhancing the overall ion transport. Recent comprehensive reviews have revealed that ion transport in polymer–ceramic composite electrolytes is strongly influenced by the interfaces between the polymer matrix and ceramic fillers, which can function as either conductive bridges or resistive barriers depending on filler microstructure, interphase chemistry, and processing routes. In particular, strategies such as ultrafast sintering of glass-ceramic powders at grain boundaries, and densification *via* hot-pressing or cold sintering have demonstrated significant reductions in grain-boundary resistance and enhancements in ionic conductivity—even in NASICON-type ceramics.^27–30^

In this study, we propose and investigate a novel amorphous inorganic/polymer composite solid electrolyte aimed at mitigating grain boundary resistance—a major drawback of conventional inorganic electrolytes—while maintaining high flexibility and formability. Ultimately, this is achieved by integrating amorphous LAGP with PEO. We elucidate the fundamental ion transport mechanisms through thermal and electrochemical characterization, and to further explore the bulk and interfacial behaviours, we employ synchrotron-based spectroscopy. This work contributes to the development of next-generation solid electrolytes with enhanced performance for high-safety, high-energy-density battery systems.

## Experimental

### Sample preparation

All sample preparations were conducted in an argon-filled glovebox (O_2_ concentration <10 ppm, dew point <193 K, Miwa Manufacturing Co., Ltd) to prevent exposure to moisture and oxygen. A polyether macromonomer, P(EO/PO) (ethylene oxide : propylene oxide = *ca.* 8 : 2, *M*_w_ ≈ 8000, TA-210, Dai-ichi Kogyo Seiyaku Co.), lithium bis(trifluoromethanesulfonyl)imide (LiTFSA; Solvay Co., Ltd), LAGP powders (Toshima Mfg Co., Ltd), a photoinitiator (*i.e.*, DMPA), and were dissolved in acetonitrile, using amber bottles to shield the solution from visible light. The concentration of LiTFSA was fixed at [Li]/[O] = 0.10, relative to the molar amount of ether oxygen in the P(EO/PO).

**Fig. 1:**
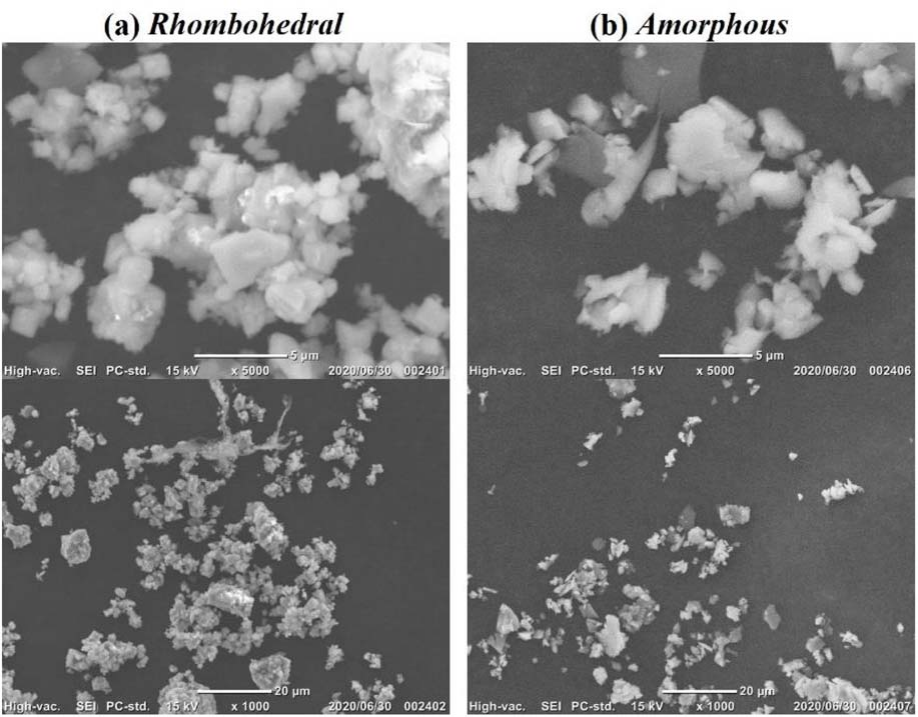
SEM images of (a) rhombohedral LAGP and (b) amorphous LAGP powders.

**Fig. 2:**
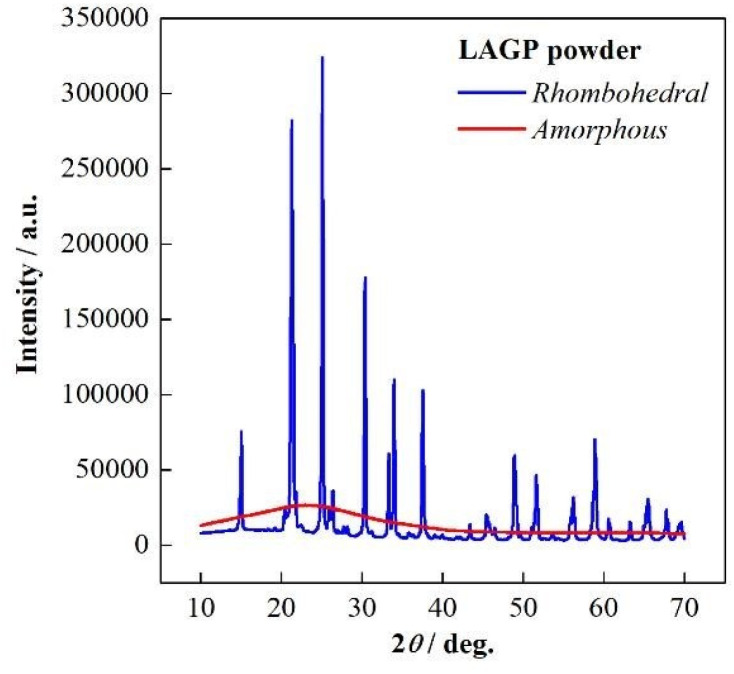
XRD patterns of rhombohedral LAGP (blue) and amorphous LAGP (red) powders.

Two types of LAGP were used: amorphous LAGP (LAGP(A)) and rhombohedral LAGP (LAGP(R)). The morphology and crystallinity of these powders were characterized by scanning electron microscopy (SEM) and X-ray diffraction (XRD) (Fig. 1 and 2, respectively). The particle sizes ranged from 10^−5^ to 10^−6^ m. The amount of LAGP was varied from 0 to 300 wt% relative to the mass of P(EO/PO). Following the addition of DMPA (0.1 wt% relative to P(EO/PO)), the resulting mixtures were vacuum-dried for 12 h to remove residual acetonitrile. The viscous slurry was then cast between two glass plates separated by 0.5 mm Teflon^®^ spacers and cured *via* UV irradiation for 5 min to initiate radical polymerization.

### Thermophysical properties of polyether/LAGP hybrid electrolytes

The glass transition temperatures (*T*_g_) of the hybrid electrolytes were measured by differential scanning calorimetry (DSC; Thermo Plus EVO2 DSC8231, Rigaku) under a nitrogen atmosphere. Electrolyte films were sealed in aluminum pans inside the glovebox to avoid atmospheric exposure. DSC measurements were conducted in the temperature range of 173.15 to 473.15 K, at a heating rate of 10 K min^−1^. The *T*_g_ values were determined as the middle point of the step change in heat capacity observed in the thermograms.

### Electrochemical measurements of polyether/LAGP hybrid electrolytes

Electrochemical impedance spectroscopy (EIS) was performed to evaluate the ionic conductivity using symmetric cells equipped with either blocking or non-blocking electrodes. Two types of cells were assembled:

Type (a): [SUS|Electrolyte|SUS]—electrolyte films were punched into 12 mm discs and sandwiched between stainless-steel (SUS) electrodes (12 mm diameter). The cells were assembled inside the glovebox and sealed to prevent air exposure. Impedance spectra were recorded over the frequency range of 200 kHz to 50 mHz with an applied AC voltage of 100 mV, at temperatures above 303.15 K. Prior to measurement, samples were thermally equilibrated for 1.5 h at each temperature.

Type (b): [Li|Electrolyte|Li]—to assess the effect of grain boundary resistance, electrolyte films (18 mm diameter) were placed between lithium metal electrodes (16 mm diameter) and sealed in 2032-type coin cells within the glovebox. EIS measurements were conducted in the 200 kHz to 10 mHz frequency range under 100 mV AC amplitude at 278.15 K, following 1.5 h of thermal equilibration.

### High-energy X-ray total scattering experiments

To investigate grain boundary effects from a molecular-level perspective, structural analyses were conducted by performing high-energy X-ray total scattering (HEXTS) measurements. Three samples were prepared in borosilicate glass capillaries: (1) polyether electrolyte without LAGP, (2) polyether/LAGP(A) hybrid electrolyte containing 150 wt% LAGP, and (3) LAGP(A) powder. For samples (1) and (2), acetonitrile was included during loading and then removed *via* vacuum drying for 12 h, followed by 5 min UV-induced radical polymerization. Sample (3) consisted of LAGP(A) powder alone, sealed in a 2 mm capillary tube.

HEXTS measurements were conducted at room temperature on the BL04B2 beamline at SPring-8 (JASRI, Japan), employing monochromatic X-rays (61.4 keV) generated using a Si(220) monochromator. The raw scattering data were corrected for absorption, polarization, and incoherent scattering to obtain the coherent scattering intensity [*I*_coh_(*q*)]. The experimental structure factor [*S*^exp^(*q*)] per stoichiometric volume was computed as follows:

1 *S*^exp^(*q*) = ((*I*_coh_(*q*) − ∑*n*_i_*f*_i_(*q*)^2^)/(∑*n*_i_*f*_i_(*q*))^2^) + 1

where *n*_i_ and *f*_i_(*q*) denote the number and atomic scattering factor of atom i, respectively. The radial distribution function (*G*^exp^(*r*)) was obtained by inverse Fourier transformation:2
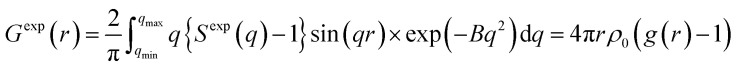
where *ρ*_0_ is the number density, *B* is the damping factor (0.008 Å^2^), and *q*_max_ was set to 23 Å^−1^.

## Results and discussion

### Thermal properties of the composite solid electrolytes

**Fig. 3:**
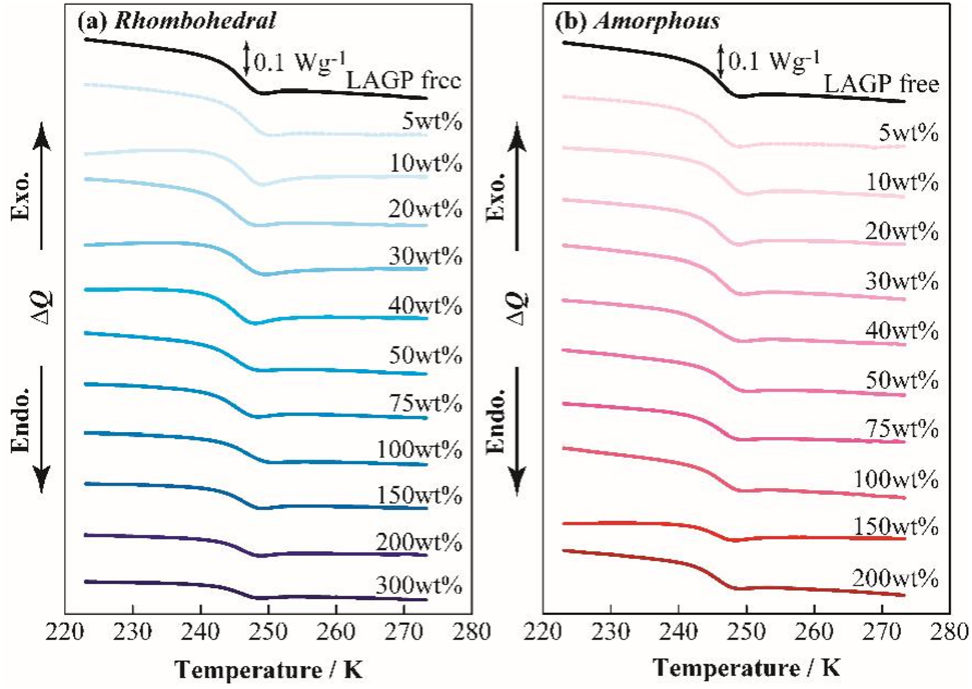
DSC thermograms of polyether-based hybrid electrolytes containing (a) rhombohedral LAGP and (b) amorphous LAGP.

Fig. 3 presents the DSC thermograms of the polyether/LAGP composite solid electrolytes (a: LAGP(R), b: LAGP(A)). The vertical axis represents the heat capacity normalized per gram of electrolyte (W g^−1^). For all compositions, a glass transition temperature (*T*_g_), corresponding to the transition from a glassy to a rubbery state, was observed. The *T*_g_ was approximately 245 K for all samples, with no significant shift as the LAGP content increased, suggesting that the incorporation of LAGP into the polyether matrix has minimal thermal impact. The change in heat capacity associated with the glass transition decreased with the increasing LAGP content, which can be attributed to the reduced proportion of polyether in the total electrolyte mass.

### Temperature dependence of the ionic conductivity

**Fig. 4:**
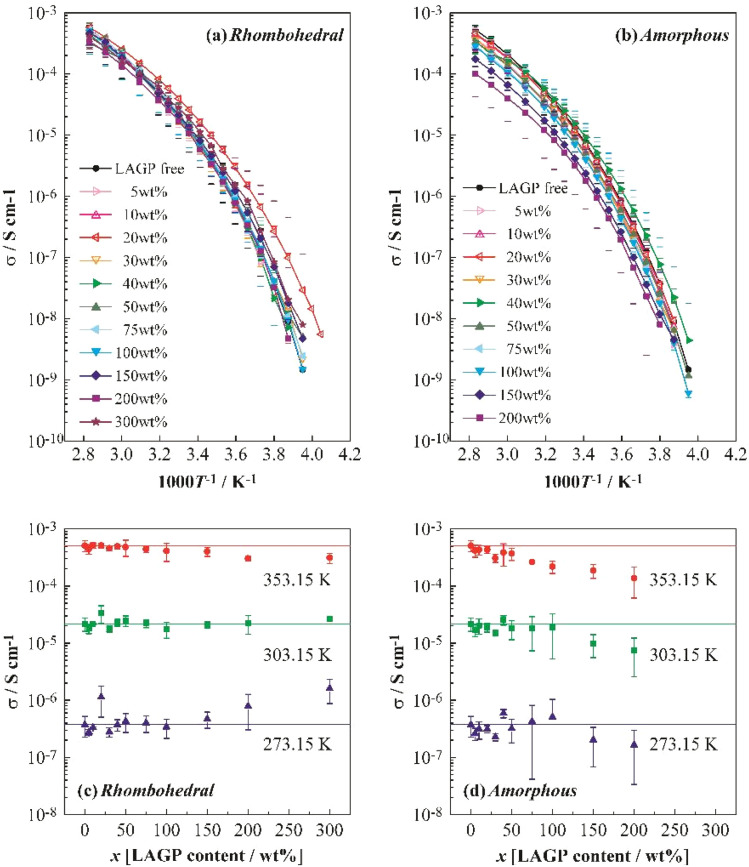
Temperature dependence of the ionic conductivity of polyether/LAGP hybrid electrolytes incorporating (a) rhombohedral and (b) amorphous LAGP, LAGP content dependence of the ionic conductivity of polyether/LAGP hybrid electrolytes incorporating (c) rhombohedral and (d) amorphous LAGP at 273.15, 303.15 and 353.15 K, respectively.

Fig. 4(a and b) shows Arrhenius plots of the ionic conductivity (*σ*) obtained for the polyether/LAGP composite solid electrolytes *via* AC impedance spectroscopy (a: LAGP(R), b: LAGP(A)). Each measurement was repeated at least three times, and the plots correspond to representative data exhibiting the median conductivity observed at 298 K. Error bars indicate the maximum and minimum values. Fig. 4(a) reveals no significant difference in *σ* between the LAGP-free polyether electrolyte and the polyether/LAGP(R) composite electrolytes. In contrast, Fig. 4(b) shows a decrease in *σ* with the increasing LAGP(A) content, likely due to the relatively low conductivity of LAGP(A). AC impedance measurements of LAGP(R) and LAGP(A) pellets (thickness: 0.5 mm, diameter: 12 mm) revealed no measurable conductivity for LAGP(A), confirming its poor ionic transport properties. Therefore, the reduction in *σ* for the polyether/LAGP(A) composites is attributed to the intrinsically low *σ* of LAGP(A).

Fig. 4(c and d) illustrate the dependence of *σ* on LAGP content at different temperatures (c: LAGP(R), d: LAGP(A)). The LAGP-free polyether electrolyte exhibited average *σ* values of 5.1 × 10^−4^, 2.2 × 10^−5^, and 3.7 × 10^−7^ S cm^−1^ at 353.15, 303.15, and 273.15 K, respectively. The LAGP(R) 100 wt% composite electrolyte showed corresponding *σ* values of 4.1 × 10^−4^, 1.8 × 10^−5^, and 3.4 × 10^−7^ S cm^−1^, all slightly lower than those of the LAGP-free sample. However, the *σ* difference decreased with the decreasing temperature. The LAGP(A) 100 wt% composite exhibited *σ* values of 2.2 × 10^−4^, 1.9 × 10^−5^, and 5.1 × 10^−7^ S cm^−1^ at the same temperatures. Notably, the *σ* of LAGP(A) was lower than that of the LAGP-free electrolyte at 353.15 and 303.15 K, but higher at 273.15 K. These results suggest that the incorporation of LAGP mitigates the decrease in *σ* at lower temperatures, thereby altering the temperature dependence of conductivity.

In general, LAGP(A) exhibits low *σ* and is expected to have lower conductivity than the polymer above a certain temperature. Given that charge carriers preferentially migrate through highly conductive phases, it is conceivable that Li^+^ ions avoid the LAGP(A) phase and migrate through the polyether matrix instead. If this is the case, the LAGP(A) particles would act as resistive obstacles, lengthening the conduction pathways and increasing resistance. However, Fig. 4 shows a suppressed decrease in *σ* at lower temperatures, indicating a change in the temperature dependence of conductivity. Given that polyether electrolytes typically show high temperature dependence, LAGP, which exhibits lower dependence, may facilitate higher *σ* at lower temperatures. The use of LAGP powders with particle sizes below 15 μm is also expected to reduce resistance compared with the use of sintered pellets. Takada *et al.* reported that glassy electrolytes exhibit lower grain boundary resistance than crystalline ceramics and that dispersing LTP particles in a glassy matrix can reduce grain boundary resistance if the interfacial resistance is low.^31^ Thus, Li^+^ ions may still migrate through the amorphous LAGP(A) phase. The results in Fig. 4 suggest that Li^+^ conduction through amorphous LAGP(A) is plausible, particularly at low temperatures.

### Impedance spectra as a function of LAGP structure and composition

**Fig. 5:**
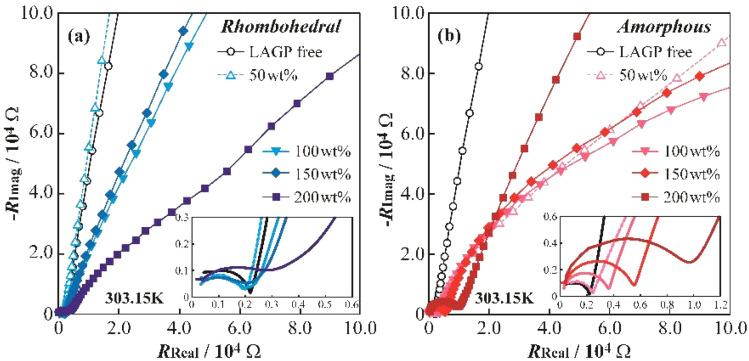
Impedance spectra of [SUS/electrolyte/SUS] symmetric cells employing polyether-based hybrid electrolytes at 303.15 K: (a) rhombohedral LAGP and (b) amorphous LAGP.

Fig. 5 shows Nyquist plots obtained from AC impedance measurements of symmetric cells [SUS (blocking electrode)|LAGP composite solid electrolyte|SUS] at 303.15 K (a: LAGP(R), b: LAGP(A)). The LAGP-free polyether electrolyte exhibited a single symmetric semicircle, attributed to the bulk resistance of the electrolyte. In Fig. 5(a), the polyether/LAGP(R) composites with 100 and 150 wt% LAGP displayed distorted semicircles, indicating the presence of additional resistance components, potentially arising from LAGP(R) particles and their interfaces with the polymer matrix. The LAGP(R) 200 wt% composite showed an even more distorted arc, suggesting that excessive LAGP(R) content may lead to the formation of continuous LAGP-rich phases within the electrolyte.

In contrast, the LAGP(A) composites in Fig. 5(b) exhibited nearly symmetric semicircles across all compositions, consistent with the bulk resistance attributed to the polymer matrix. While the resistance increased with the increasing LAGP(A) content, no significant distortion was observed, implying minimal interfacial or grain boundary resistance. This may be ascribed to the difficulty in detecting the small resistance contribution of the amorphous LAGP phase, even in high-LAGP-content composites. Takada *et al.* stated that grain boundary resistance in amorphous powders can be negligible, as evidenced by similar activation energies between pressed powders and sintered bodies.^32^ However, it is important to consider the possibility that, at 303.15 K, Li^+^ ions may not be conducting through the LAGP(A) phase, and the observed resistance increase may result from longer conduction paths due to LAGP particle avoidance.

### Evaluation of the grain boundary effects using non-blocking electrodes

**Fig. 6:**
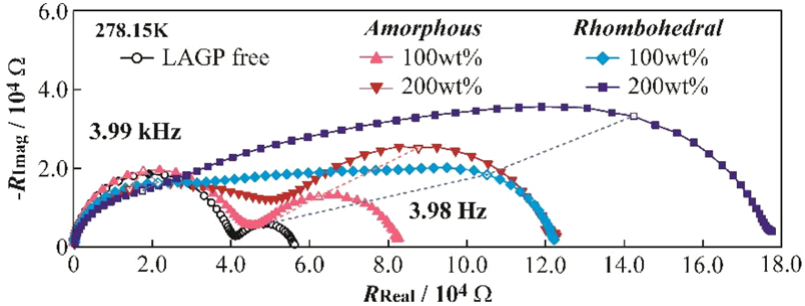
Impedance spectra of [Li/electrolyte/Li] symmetric cells employing polyether-based hybrid electrolytes at 273.15 K.

Fig. 6 presents impedance spectra measured using non-blocking Li metal electrodes. The chemical stability of LAGP against Li metal was confirmed by the absence of time-dependent changes in the spectra. The polyether electrolyte exhibited two distinct resistance components, attributed to bulk resistance at high frequency and Li/electrolyte interfacial resistance at low frequency. Clear spectral differences were observed between the LAGP(A) and LAGP(R) composites. The LAGP(A) composite showed at least two resistance components, whereas the crystalline LAGP(R) composite exhibited multiple unresolved arcs, suggesting the presence of several resistance contributions. Although the resistance values showed significant variation, all compositions exhibited characteristic spectral shapes. In previous work, we identified the grain boundary resistance in LLZO/polyether composites as an intermediate resistance between bulk and interfacial components. Applying a similar rationale, the multiple arcs observed in crystalline LAGP(R) composites are likely due to relatively high grain boundary resistance. The aspect ratios of the arcs in the LAGP(R) 100 and 200 wt% composites were calculated to be 5.5–6.5 and 4.9–5.9, respectively. A single ideal semicircle has an aspect ratio of 2, while two equal-sized semicircles have a maximum of ∼4. Therefore, the LAGP(R) composites contain more than two distinct resistance components, with higher aspect ratios correlating with higher LAGP(R) contents, further supporting the presence of grain boundary resistance.

The LAGP(A) 200 wt% composite exhibited a deformed arc on the high-frequency side, indicating the emergence of a new resistance component. This may be due to increased particle contact frequency or larger interfacial areas between LAGP and the polymer. Impedance measurements of LAGP(A) and LAGP(R) pellets revealed that the composite spectra lacked resistance components attributable to bulk LAGP(A), suggesting some limited Li^+^ conduction through these particles. While this does not definitively confirm Li^+^ conduction through the LAGP(A) phase, the results imply that the use of amorphous solid electrolytes can effectively suppress resistance contributions from inorganic particles in composite electrolytes. This highlights the potential advantage of using amorphous ceramics in the design of organic–inorganic hybrid solid electrolytes.

### Structural analysis using synchrotron radiation

**Fig. 7:**
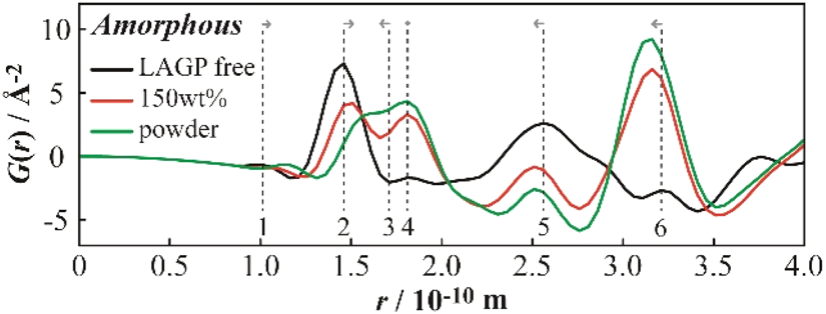
HEXTS measurement results of amorphous LAGP.

HEXTS measurements were performed at BL04B2 of SPring-8 to investigate the local structure of the composite electrolytes. The resulting pair distribution functions (PDFs), reflecting the atomic pair distances, are shown in Fig. 7. Measurements were conducted on the polyether electrolyte, LAGP(A) 150 wt% composite electrolyte, and LAGP(A) powder. All samples displayed periodic features in their PDFs. Notably, the PDF of the LAGP(A) composite was intermediate between those of the individual components, indicating favourable interfacial interaction and close structural correlation between the polymer and the inorganic phase. These results also demonstrate the applicability of HEXTS as a powerful technique for probing local structures in polymer–amorphous ceramic composites. Therefore, practical implementations of polymer/ceramic composite films have demonstrated improved stability at elevated voltage and enhanced lithium metal compatibility, as well as tuneable mechanical–electrochemical trade-offs *via* composition and processing control. The current results—showing suppressed interfacial resistance under non-blocking conditions and favourable structural integration *via* HEXTS—are fully consistent with these established hybrid electrolyte behaviours.^27,29^ Further studies using solid-state NMR, Raman spectroscopy, and X-ray photoelectron spectroscopy (XPS) are expected to provide complementary insights into the local and interfacial structures of the grain boundaries, thereby supporting the findings obtained from the HEXTS analysis.

## Conclusions

In this study, we investigated the effects of incorporating either crystalline or amorphous LAGP into a polyether-based polymer matrix to fabricate composite solid electrolytes. DSC revealed that the addition of LAGP did not significantly alter the thermal transitions of the host polymer, indicating that the inorganic filler exerts minimal influence on the segmental motion of the polymer chains. Ionic conductivity measurements demonstrated that crystalline LAGP (LAGP(R)) led to a modest decrease in conductivity, as expected, whereas amorphous LAGP (LAGP(A)) exhibited a more complex temperature-dependent behaviour. Notably, the incorporation of LAGP(A) effectively mitigated the sharp decline in ionic conductivity at lower temperatures, suggesting possible Li^+^ transport through the amorphous ceramic phase. EIS revealed that composites containing crystalline LAGP suffered from pronounced grain boundary resistance, as evidenced by distorted and multi-arc Nyquist profiles. In contrast, composites incorporating amorphous LAGP exhibited suppressed interfacial and grain boundary resistance, particularly when non-blocking electrodes were employed. These findings underscore the intrinsic advantages of amorphous ceramics in polymer composite systems designed for solid electrolytes, offering smoother ion-conduction pathways and improved interfacial compatibility. Finally, HEXTS measurements confirmed a favourable structural integration between the polymer matrix and the amorphous LAGP phase. The pair distribution function of the composite electrolyte was intermediate between those of its constituents, signifying a high degree of atomic-level mixing and intimate phase interaction. Collectively, our results highlight the critical role of the microstructure of inorganic fillers in dictating ion transport properties within composite solid electrolytes. In particular, the use of amorphous solid electrolytes presents a promising strategy for minimizing interfacial resistance and enhancing low-temperature performance in next-generation all-solid-state lithium batteries.

## Conflicts of interest

There are no conflicts to declare.

## Supplementary Material

RA-015-D5RA07453C-s001

## Data Availability

The data supporting this article have been included as part of the supplementary information (SI). Supplementary information is available. See DOI: https://doi.org/10.1039/d5ra07453c.

## References

[cit1] Perrin M., Saint-Drenan Y. M., Mattera F., Malbranche P. (2005). J. Power Sources.

[cit2] Dunn B., Kamath H., Tarascon J.-M. (2011). Science.

[cit3] Yoshino A., Tsubata T., Shimoyamada M., Satake H., Okano Y., Mori S., Yata S. (2004). J. Electrochem. Soc..

[cit4] Armand M., Tarascon J.-M. (2008). Nature.

[cit5] Goodenough J. B., Kim Y. (2010). Chem. Mater..

[cit6] Fergus J. W. (2010). J. Power Sources.

[cit7] Sun C., Liu J., Gong Y., Wilkinson D. P., Zhang J. (2017). Nano Energy.

[cit8] Balakrishnan P. G., Ramesh R., Prem Kumar T. (2006). J. Power Sources.

[cit9] Long L., Wang S., Xiao M., Meng Y. (2016). J. Mater. Chem. A.

[cit10] Inaguma Y., Liquan C., Itoh M., Nakamura T., Uchida T., Ikuta H., Wakihara M. (1993). Solid State Commun..

[cit11] Park H., Jung K., Nezafati M., Kim C.-S., Kang K. (2016). ACS Appl. Mater. Interfaces.

[cit12] Janek J., Zeier W. G. (2016). Nat. Energy.

[cit13] Sun Y., Suzuki K., Hori S., Hirayama M., Kanno R. (2017). Chem. Mater..

[cit14] Breuer S., Prutsch D., Ma Q., Epp V., Preishuber- Pflügl F., Tietz F., Wilkening M. (2015). J. Mater. Chem. A.

[cit15] Nishimoto A., Agehara K., Furuya N., Watanabe T., Watanabe M. (1999). Macromolecules.

[cit16] Acosta J. L., Morales E. (1996). Solid State Ionics.

[cit17] Zhang Z., Huang Y., Gao H., Huang J., Li C., Liu P. (2020). Ceram. Int..

[cit18] Keller M., Appetecchi G. B., Kim G.-T., Sharova V., Schneider M., Schuhmacher J., Roters A., Passerini S. (2017). J. Power Sources.

[cit19] Hiraoka K., Kato M., Kobayashi T., Seki S. (2020). J. Phys. Chem. C.

[cit20] Kato M., Hiraoka K., Seki S. (2020). J. Electrochem. Soc..

[cit21] Thokchom J. S., Gupta N., Kumar B. (2008). J. Electrochem. Soc..

[cit22] Jung Y.-C., Lee S.-M., Choi J.-H., Jang S. S., Kim D.-W. (2015). J. Electrochem. Soc..

[cit23] Guin M., Indris S., Kaus M., Ehrenberg H., Tietz F., Guillon O. (2017). Solid State Ionics.

[cit24] Xiao W., Wang J., Fan L., Zhang J., Li X. (2019). Energy Storage Mater..

[cit25] Xu X., Wen Z., Wu X., Yang X., Gu Z. (2007). J. Am. Ceram. Soc..

[cit26] Du M., Liao K., Lu Q., Shao Z. (2019). Energy Environ. Sci..

[cit27] Alsaç E. P., Nelson D. L., Yoon S. G., Cavallaro K. A., Wang C., Sandoval S. E., Eze U. D., Jeong W. J., McDowell M. T. (2025). Chem. Rev..

[cit28] Sand S. C., Rupp J. L. M., Yildiz B. (2025). Chem. Soc. Rev..

[cit29] Chang H., Zhang X., Li W., Liu H., Hu H., Liu Z., Liu W., Jin Y. (2025). Next Mater..

[cit30] Lee R., Kang C., Lee J., Jin B., Kim K., Kim H., Yoon J., Lee S. (2024). NPG Asia Mater..

[cit31] Takada K., Tansho M., Yanase I., Inada T., Kajiyama A., Kouguchi M., Kondo S., Watanabe M. (2001). Solid State Ionics.

[cit32] Takada K., Kondo S. (1998). Ionics.

