# Supplementary material for: Tailoring grain boundary resistance in Li-ion conducting polymer–ceramic hybrid electrolytes based on polyether and Li1.5Al0.5Ge1.5(PO4)3
Source: RSC Adv. 2025 Oct 29;15(49):41530–6. doi: 10.1039/d5ra07453c (PMC12570871; doi:10.1039/d5ra07453c)
Supplement: RA-015-D5RA07453C-s001 [file RA-015-D5RA07453C-s001.pdf]

Supplementary Information (SI) for RSC Advances.
This journal is © The Royal Society of Chemistry 2025

Electronic Supplementary Information (ESI)

Tailoring Grain Boundary Resistance in Li-ion Conducting Polymer–Ceramic Hybrid Electrolytes Based on Polyether and $Li_{1.5}Al_{0.5}Ge_{1.5}(PO_4)_3$

Naamo Suzuki[a], Koji Hiraoka [a], Koji Ohara[b], Kenta Fujii[c], and Shiro Seki*[,a]

[a] *Graduate School of Applied Chemistry and Chemical Engineering, Kogakuin University, 2665-1 Nakano-machi, Hachioji, Tokyo, 192-0015, Japan.*

[b] *Faculty of Materials for Energy, Shimane University, 1060, Nishi-Kawatsu-Cho, Matsue, Shimane 690-8504, Japan*

[c] *Faculty of Engineering Department of Applied Chemistry Yamaguchi University, 16-1 Tokiwadai 2-chome, Ube-shi, 755-8611 Yamaguchi*

---

* Corresponding author.

Fax: +81-42-628-4568; Tel: +81-42-628-4568, E-mail: shiro-seki@cc.kogakuin.ac.jp (S. Seki)

Table S1 The measured T_gs of polyether-based hybrid electrolytes containing rhombohedral LAGP and amorphous LAGP. The T_g were determined as middle point of heat capacity change in DSC thermograms.

	T_g / K	
**LAGP content / wt%**	**Rhombohedral**	**Amorphous**
0	243.9	243.9
5	245.8	245.1
10	245.5	245.0
20	243.9	244.3
30	245.0	244.3
40	243.9	243.9
50	244.1	245.3
75	244.2	244.3
100	245.8	245.0
150	245.1	244.2
200	243.1	243.3
300	244.8	N/A

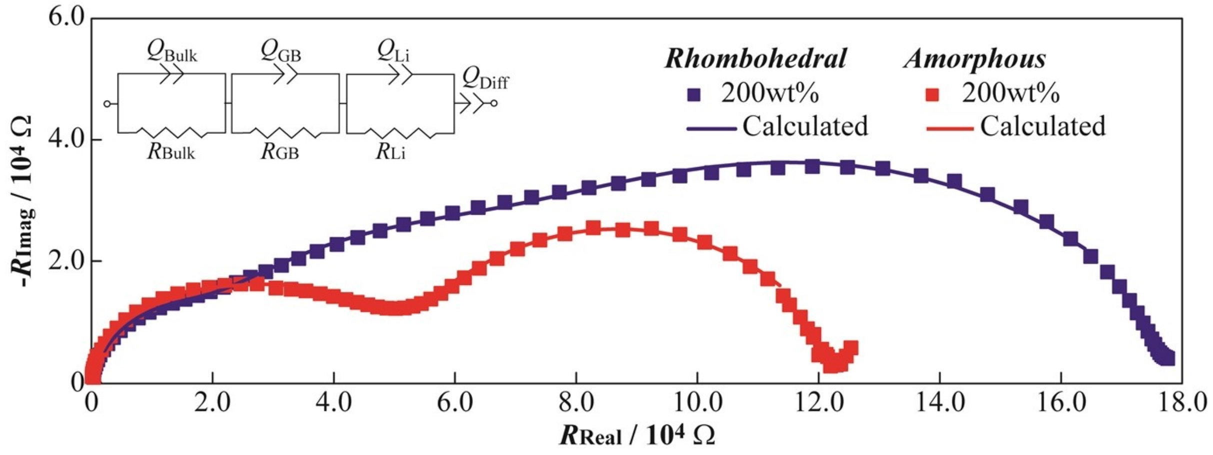

Figure S1 Impedance spectra of [Li/electrolyte/Li] symmetric cells employing polyether-based hybrid electrolytes at 273.15 K with calculated profiles using equivalent circuit. In the equivalent circuit, Q_{Bulk}, Q_{GB}, Q_{Li} and Q_{Diff} are constant-phase elements of SPE and LAGPs bulk, grain LAGPs boundary, electrolyte/Li metal interface, infinite diffusion element, and R_{Bulk}, R_{GB}, R_{Li} are direct current resistances of SPE and LAGPs bulk, LAGPs grain boundary, electrolyte/Li metal interface.
